# Magnetic resonance imaging and neurological findings in dogs with disc‐associated cervical spondylomyelopathy: a case series

**DOI:** 10.1186/s12917-021-02846-5

**Published:** 2021-04-07

**Authors:** Marília de Albuquerque Bonelli, Luciana Bignardi de Soares Brisola Casimiro da Costa, Ronaldo Casimiro da Costa

**Affiliations:** 1 Department of Veterinary Clinical Sciences, College of Veterinary Medicine, The Ohio State University, 601 Vernon Tharp St, OH 43210 Columbus, USA; 2 Department of Veterinary Preventive Medicine, College of Veterinary Medicine, The Ohio State University, 601 Vernon Tharp St, 43210 Columbus, OH USA

**Keywords:** Canine, Cervical spine, Diagnostic imaging, MRI, Wobbler syndrome

## Abstract

**Background:**

Canine cervical spondylomyelopathy can be separated into osseous and disc-associated (DA-CSM) forms. Our aim was to describe the magnetic resonance imaging (using a high-field scanner) and neurological findings in dogs with DA-CSM and investigate a relationship between these findings.

**Results:**

Sixty-three dogs were included: 60/63 (95 %) were large breeds, with Doberman Pinschers and males over-represented (70 %). Mean and median age at the time of diagnosis was 7.25 and 7.2 years (range 0.41–12 years). Chronic signs were noted in 52/63 (83 %) dogs, with proprioceptive ataxia the most common. Main site of spinal cord compression was commonly C6-7 or C5-6. Thirty-six (57 %) dogs had various sites of spinal cord compression. Most dogs younger than 6 years of age had a single affected site. Foraminal stenosis was present in 51/63 dogs (81 %). T2-weighted hyperintensity was present in 40/63 dogs (63 %). 88 % of the articular processes showed degenerative changes, which correlated strongly with intervertebral disc degeneration. Ligamentum flavum hypertrophy was seen in 38 % of dogs. No correlation was observed between neurologic signs and number of affected sites. A moderate positive correlation was observed between severity of spinal cord compression and neurologic grade (*r* 0.48; *p* < 0.001).

**Conclusions:**

DA-CSM was predominantly observed in older, male Dobermans, with lesions located in the caudal cervical vertebral region. It was also seen in dogs 3 years of age or even younger (8 %). Single compressive lesions were more common in dogs younger than 6 years of age. Many dogs had concomitant changes (e.g.: ligamentum flavum hypertrophy and foraminal stenosis). Most dogs with ligamentum flavum hypertrophy were 6 years or older. A positive correlation was observed between severity of spinal cord compression and neurologic grade, but multilevel compression was not associated with more severe neurologic signs. A very high percentage of dogs had articular process degenerative changes. Possible biomechanical or genetic relationships between degenerative changes in articular processes, ligamentum flavum, and intervertebral discs warrants further investigation.

## Background

Cervical spondylomyelopathy (CSM), also known as wobbler syndrome, affects the cervical vertebral column of dogs and causes compression of the spinal cord and/or nerve roots [1, 2] mostly in large and giant breeds [3–8].

Commonly, there are two recognized forms of CSM, which may occur separately or in conjunction: one associated with disc protrusion and the other with osseous proliferation [1]. Disc-associated CSM (DA-CSM) is a result of spinal cord and/ or nerve root compression secondary to intervertebral disc protrusion and is typically more prevalent in Doberman Pinschers and other large breeds [9, 10].

Although there are numerous studies on CSM, there are few studies focused primarily on the imaging characteristics of disc-associated CSM using magnetic resonance imaging (MRI); only one using high-field MRI in Doberman Pinschers [9], and two using low-field MRI [11, 12], the latter solely on Doberman Pinschers [12]. The imaging features of DA-CSM using high-field MRI in a large population of dogs has not been thoroughly investigated. The possible association between clinical and imaging findings have also not been studied. Our objective was to describe the MRI and neurological findings in dogs with disc-associated CSM using a high-field MRI scanner and to investigate a relationship between neurological signs and MRI findings. We also aimed to compare the findings in dogs that had one site and multiple sites of spinal cord compression. Our hypotheses were that either the severity of spinal cord compression or multilevel compressions would be associated with more severe neurologic grade.

## Results

Sixty-three (27 %) out of 232 dogs diagnosed with CSM during the study period met the inclusion criteria for the diagnosis of DA-CSM in our study. Out of these, 60/63 (95 %) were large breeds, with the majority (44/63; 70 %) being Doberman Pinschers. Other breeds included Weimaraner (8), German Shepherd (2), American Pit Bull Terrier, Basset Hound, Bernese Mountain Dog, Dalmatian, Great Dane, Husky mix, Rhodesian Ridgeback, Saluki, and Standard poodle (1 each). Mean and median age at the time of CSM diagnosis was 7.25 and 7.2 years, respectively (range 0.41–12 years). There were 5/63 (8 %) dogs that were 3 years of age or younger. The majority of dogs (43/63; 68 %) were 6 years or older. Forty-four out of 63 (70 %) dogs were male and 19/63 (30 %) were female, though gender was not statistically significant for the investigated parameters.

Regarding presentation, 52/63 (83 %) dogs had a chronic presentation (5/52 reported acute worsening of chronic signs), while 11/63 (17 %) had an acute manifestation of the neurologic signs. Duration of clinical signs at the time of diagnosis varied between 0.03 and 36 months (mean 6 months, median 2 months).

Forty-nine out of 60 (78 %) dogs had proprioceptive ataxia, and 36/63 (58 %) had tetraparesis at the time of diagnosis (5/27 were non-ambulatory – 19 %). A total of 31/63 (49 %) dogs showed signs of cervical hyperesthesia upon neurologic examination (17 of which had cervical pain as a historical finding). Nine dogs (9/63; 14 %) had cervical hyperesthesia as the only presenting complaint. Neurologic grade for the dogs at the time of diagnosis was a mean of 2.9 and median of 3 (range 1 to 5).

**Fig. 1:**
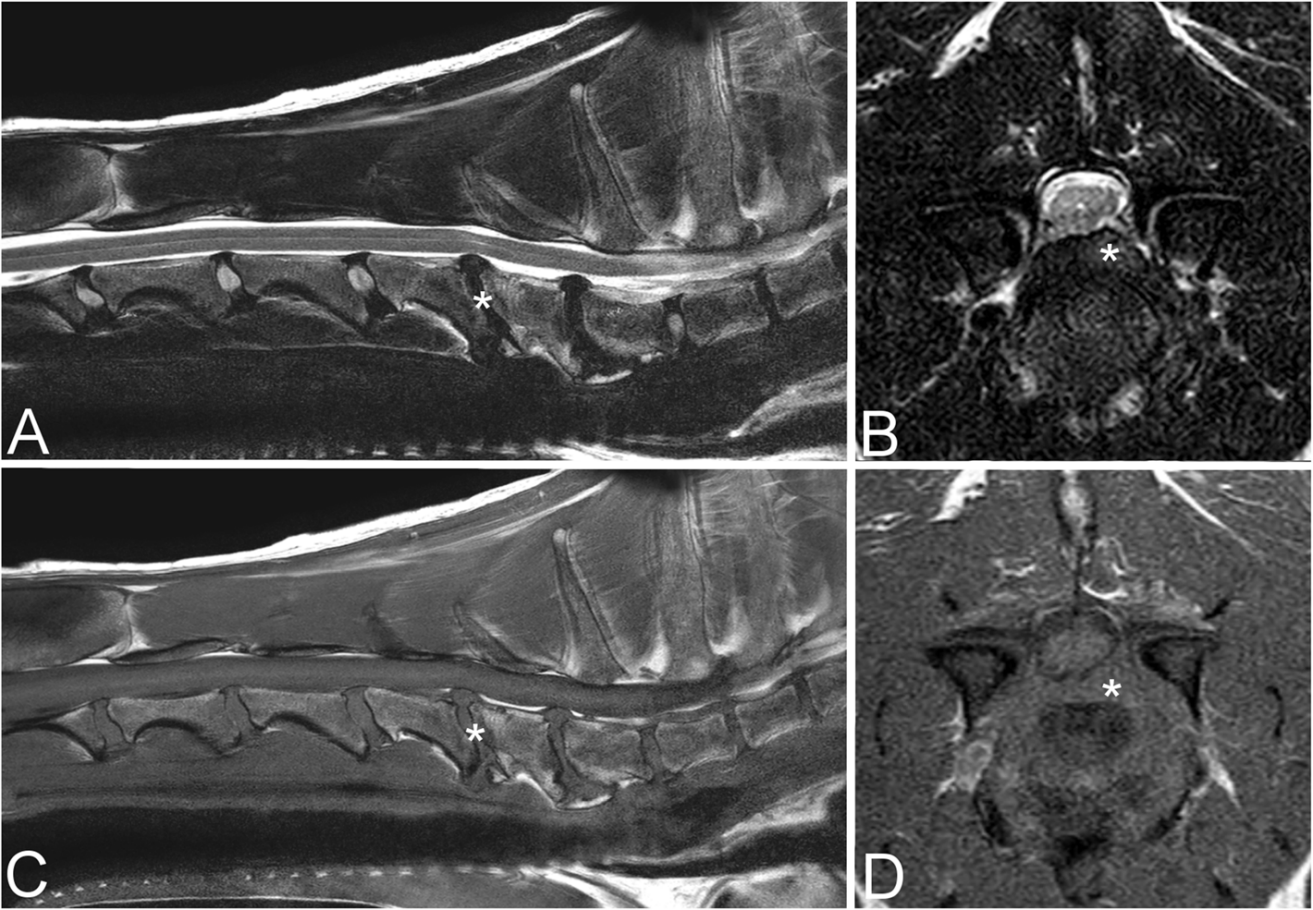
Sagittal (**a**) and transverse (**b**) T2W and sagittal (**c**) and transverse T1W magnetic resonance imaging of an 11-year-old Weimaraner diagnosed with cervical spondylomyelopathy due to spinal cord compression from intervertebral disc protrusion at C5-6 (asterisk). Note varying degrees of intervertebral disc protrusions at other sites

The site of principal spinal cord compression was C6-7 in 33/63 (52 %) dogs, C5-6 in 24/63 (38 %) dogs, and C4-5 and C3-4 in 3 dogs (5 %) each. Main spinal cord compression was caused solely by intervertebral disc protrusion in 47/63 (75 %) dogs (Fig. 1), intervertebral disc protrusion and ligamentum flavum hypertrophy in 11/63 (17 %) dogs, ligamentum flavum hypertrophy in 3/63 (5 %) dogs (these dogs also had protrusion at another site), and intervertebral disc protrusion with “tipping” of the vertebral body in 2/63 (3 %) dogs.

**Fig. 2:**
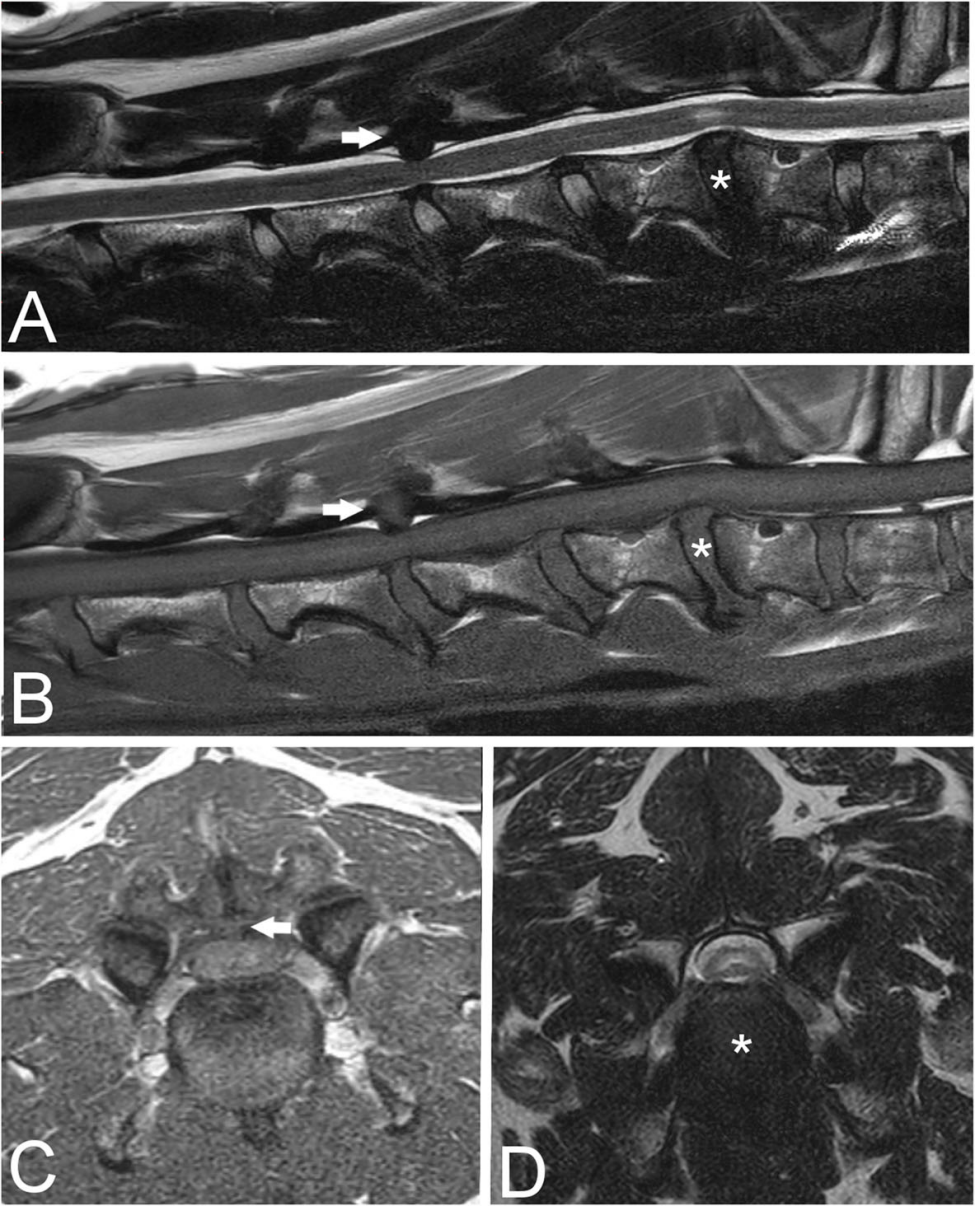
Sagittal T2-weighted (**a**) and T1-weighted (**b**) and transverse T1W (**c**) and T2W (**d**) magnetic resonance images from an 8-year-old Doberman Pinscher diagnosed with disc-associated cervical spondylomyelopathy. Spinal cord compression is observed due to intervertebral disc protrusion at C6-7 (asterisk) and intervertebral disc protrusion and ligamentum flavum hypertrophy at C4-5 (arrow). Note T2W hyperintensity of the spinal cord parenchyma at C6-7

Overall, 24/63 (38 %) dogs had concomitant ligamentum flavum hypertrophy causing spinal cord compression (Fig. 2). This was located at the same site as the intervertebral disc protrusion in 13/24 (54 %) dogs, while in 7/24 (29 %) dogs they were located at a different site and 4/24 (17 %) dogs had ligamentum hypertrophy both at the same site of intervertebral disc protrusion and at a different site.

A total of 36/63 (57 %) dogs had more than one site of spinal cord compression: 23/63 dogs (37 %) had 2 sites of spinal cord compression, 10/63 dogs (16 %) had 3 sites of compression, 2/63 dogs (3 %) had 4 sites of spinal cord compression, and 1/63 dogs (2 %) had 5 sites of spinal cord compression (Table 1). The majority (16/20, 80 %) of dogs younger than 6 years of age had a single affected site while the majority (32/43, 53 %) of dogs that were 6 years or older had multiple sites affected. Only 3/20 (15 %) dogs younger than 6 years had ligamentum flavum hypertrophy, while 21/43 (49 %) of the dogs 6 years or older had ligamentum flavum hypertrophy.

**Table 1:** Spinal cord compression and SCSC in dogs with multiple sites of DA-CSM

Dogs (n)	Spinal cord compression sites (n) per dog	Sites of spinal cord compression	Sites with SCSC (n)
7	2	C5-6, C6-7	C6-7 (5)
5	2	C5-6, C6-7	C5-6 (1)
5	3	C4-5, C5-6, C6-7	C5-6 (5)
4	2	C4-5, C6-7	C6-7 (4)
3	3	C4-5, C5-6, C6-7	C6-7 (2)
3	2	C4-5, C5-6	C5-6 (2)
2	2	C2-3, C5-6	C5-6 (1)
1	5	C1-2, C3-4, C4-5, C5-6, C6-7	C5-6 (1)
1	4	C3-4, C4-5, C5-6, C6-7	C6-7 (1)
1	4	C2-3, C4-5, C5-6, C6-7	C6-7 (1)
1	3	C3-4, C5-6, C6-7	C6-7 (1), C5-6 (1)
1	3	C3-4, C4-5, C6-7	0
1	3	C2-3, C5-6, C6-7	C6-7 (1)
1	2	C2-3, C3-4	0

Main sites of spinal cord compression are underlined

*n* number, *SCSC* spinal cord signal changes

When comparing dogs with single vs. multiple affected sites, dogs with ligamentum flavum hypertrophy more commonly had multiple sites of spinal cord compression (*p* < 0.001). No other statistical significance was observed. Of note, whether dogs had single or multiple sites affected had no relationship detected with neurologic grade or signal changes in the spinal cord parenchyma.

Statistically significant correlations between neurologic and imaging parameters were a moderate positive correlation between severity of spinal cord compression and neurologic grade (*r* 0.48; *p* < 0.001), between age and number of protruded discs (*r* 0.54; *p* < 0.001), between age and number of intervertebral discs with total degeneration (*r* 0.47; *p* < 0.001), and between age and spondylosis (*r* 0.48; *p* < 0.001). A moderate correlation was also observed between the number of protruded discs causing spinal cord compression and number of intervertebral discs with signs of total degeneration (*r* 0.45; *p* < 0.001) and presence of ligamentum flavum hypertrophy (*r* 0.46; *p* < 0.001).

Regarding comparison between neurologic grades, a significant difference was observed (*p* < 0.001). Dogs with spinal cord compression with a severity score of 3 (18 dogs) had a neurologic grade of 3 or higher (9/18 were grade 3, 6/18 were grade 4, 3/18 were grade 5). Dogs with spinal cord compression with a severity score of 2 (27 dogs) mostly had a neurologic grade between 2 and 4 (9/27 were grade 4, 7/27 were grade 2, and 7/27 were grade 3). As for dogs with spinal cord compression with a severity of 1 (18 dogs), the majority had neurologic grades of 1 and 2 (7/18 were grade 2 and 6/18 were grade 1). Of note, there was one dog with spinal cord compression severity score of 1 and one dog with severity score of 2 that presented with neurologic grade of 5. The former had an acute presentation and the latter had acute worsening of chronic signs.

A significant difference was seen when comparing grouped neurologic scores for severity of spinal cord compression (*p* < 0.05). The majority of dogs with spinal cord compression severity score of 1 (15/18) and 2 (17/27) had neurologic grade 1 to 3 (Group A).

Signs indicative of spinal cord atrophy were observed in 20/63 dogs. Spinal cord signal changes with T2-weighted (T2W) hyperintensity were present in 40/63 dogs (63 %), mostly located at C6-7 (25/40; 63 %) and C5-6 (14/40; 35 %). Of these, 8/40 (20 %) dogs also had T1-weighted (T1W) hypointensity, all located at C6-7 in the same site as the T2W hyperintensity. The remaining 32/40 dogs (80 %) had T2W hyperintensity which appeared as isointense on T1W images. The T2W spinal cord hyperintensities seen in all dogs were focal, centered and restricted to the intervertebral disc space (typically not extending beyond either the cranial and/or caudal endplates), commonly affecting mostly the central portion of the spinal cord, and without concurrent evidence of spinal cord swelling.

Most dogs (31/40; 78 %) with T2W hyperintensity had neurologic grade 3 or higher. When comparing grouped neurologic scores, 17/22 dogs (77 %) in score group B (grades 4 and 5) and 22/41 (54 %) dogs in score group A (grades 1–3) had T2W hyperintensity signal changes, but no statistical significance was observed. There was a moderate correlation between T2W hyperintensity and neurologic grade (*r* 0.49; *p* < 0.05) and weak correlation between T2W hyperintensity and severity of spinal cord compression (*r* 0.30, *p* < 0.05). There was also a weak correlation between T1W hypointensity and duration of clinical signs (*r* 0.32, *p* < 0.05), but no correlation with neurologic grade.

As for correlation between degenerative changes of the intervertebral disc and articular processes, a very strong positive correlation was observed between the number of intervertebral discs with total degeneration and the number of articular processes with irregular surface and signs of subchondral sclerosis (*r* 0.90; *p <* 0.05). A very strong correlation was also found between the number of intervertebral discs with total degeneration and the total number of articular process surfaces with any signs of degenerative changes (*r* 0.84; *p* < 0.05) and between the total number of intervertebral discs with signs of degenerative changes and the number of articular processes with irregular surface and signs of subchondral sclerosis (*r* 0.81; *p* = 0.05).

Sixty-two out of 63 dogs (98 %) had intervertebral disc degeneration in at least one site between C2 and T1 (total of 287 discs affected out of 378). Overall, 174/287 (61 %) intervertebral discs were classified as having partial degeneration and 113/287 (39 %) discs were classified as having total degeneration. Forty-six out of 63 dogs (73 %) had at least four discs with signs of degeneration: 25/63 (40 %) dogs had 6 discs; 11/63 (17 %) had 5 discs; and 10/63 (16 %) had 4 discs with signs of degeneration.

A total of 31/63 (49 %) dogs had changes in intervertebral endplates: 22/31 (71 %) dogs had T1W and T2W hypointense changes, 7/31 (23 %) dogs had T1W and T2W hyperintense changes, and 2/31 (6 %) dog had T1W and T2W hypointense changes in one site and T1W and T2W hyperintense changes in another. These changes were located at C6-7 in 17/25 (68 %) dogs and was at the same site as intervertebral disc degeneration in all 25 dogs.

Foraminal stenosis unrelated to bony proliferation of the articular processes was present in some degree in 51/63 dogs (81 %) in at least one location. A weak correlation was noted between tetraparesis and the presence of foraminal stenosis (*r* 0.26; *p* = 0.04). Spondylosis was noted in 33/63 dogs (52 %), and a moderate positive correlation was observed between age and spondylosis (*r* 0.48; *p* < 0.001).

Regarding degenerative changes to the articular processes, 56/63 (89 %) dogs had some degree of subchondral sclerosis with reduced or absent amount of synovial fluid noted. A total of 319 out of 378 (84 %) intervertebral disc spaces between C2 and T1 were available for evaluation. Of these, 279/319 (88 %) had some degree of subchondral sclerosis and 280/319 (88 %) had evidence of reduced or absent synovial fluid on MRI.

## Discussion

In this study, we report the first high-field MRI case series of dogs with disc-associated CSM. Most of the dogs in this series were 6 years of age or older (68 %), primarily Doberman Pinschers (70 %), male (70 %), and had a chronic history of progressive signs (83 %). These findings are in alignment with observations from other reports [10–14]. We did observe DA-CSM also affecting 8 % of young dogs, which was infrequently reported [12, 13]. As expected in DA-CSM, compressive lesions were centered in the caudal cervical vertebral region (C6-7, C5-6) in 90 % of the dogs. Proprioceptive ataxia was the most common clinical sign (78 %), followed by paresis (58 %) and cervical pain (49 %).

Regarding whether severity of compression of the spinal cord would be associated with a worse neurologic grade, a moderate positive correlation was observed between severity of spinal cord compression and neurologic grade. This may suggest that severity of spinal cord compression is at least partly responsible for presenting neurologic grade in DA-CSM. Previous studies with osseous-associated CSM [7] and DA-CSM [15] have not found a correlation between severity of compression and neurologic score. The latter study was done using a low-field MRI using 21 dogs [15].

Studies looking at intervertebral disc herniations in dogs have also tried investigating a correlation between degree of spinal cord compression and neurological grade [16–19]. Despite a noted trend of increasing spinal cord compression seen on MRI and more severe neurologic grade in dogs with acute thoracolumbar disc herniations [18], a correlation between these two variables has not been confirmed [16]. A correlation was, however, observed between presenting neurologic grade of dogs with disc extrusion in the cervical region [19]. This discrepancy may in part be explained due to the anatomical differences between the cervical and thoracolumbar regions of the vertebral column [19]. Also, factors such as initial concussive force of the extrusion and length of the compression likely also play important roles in acute intervertebral disc extrusions [18, 19].

Although in DA-CSM, concussive force and length of the compression do not commonly play a role, other equally complex forces may help explain the discrepancies found when dealing with DA-CSM [15]. The presence of dynamic lesions, for instance, may lead to divergences between spinal cord compression in neutral position and maximum spinal cord compression, since dynamic or kinematic MRI has shown that extension and flexion of the cervical vertebral column can affect the degree of spinal cord compression [20].

Most of the dogs in this study (63 %) had spinal cord signal changes in the form of T2W hyperintensity. This percentage is similar to the overall average (56.5 %) published for dogs with either form of CSM [3, 8, 21, 22] and that reported specifically in dogs with DA-CSM [12]. T1W hypointensity was not as prevalent, and was only observed in 20 % of dogs with spinal cord signal changes. This percentage was higher than previously described for DA-CSM using a low-field MRI [15] and for osseous-associated CSM [8]. The fact that all dogs were imaged with a 3 Tesla MRI in our study could account for the difference.

Correlations observed for T2W hyperintensity with neurologic grade and with severity of spinal cord compression, as well as between T1W hypointensity and duration of clinical signs seem to be in line with previous suppositions, where T2W hyperintensity would be associated with clinical relevance of the lesion [15] and T1W hypointensity with chronicity [23]. Spinal cord signal changes, as represented by an association of T2W hyperintensity and T1W hypointensity, have been associated with gray matter changes such as motor neuron loss and necrosis and have been considered indicative of a worse prognosis [24–26]. Further studies are still needed to fully understand how the presence of spinal cord signal changes may be related to neurological status and prognosis in dogs.

As for whether dogs with multiple sites of spinal cord compression would be more severely affected, but this was not confirmed in the present study. In dogs with osseous-associated CSM, a correlation between number of affected sites and neurological signs has also not been observed [7]. We found that the majority of dogs (57 %) had more than one site of spinal cord compression, with 37 % of these having two sites of compression. It is possible that the presence of less severe sites of spinal cord compression would not sufficiently worsen the patient’s neurological presentation.

A total of 38 % dogs had concomitant ligamentum flavum hypertrophy causing some degree of spinal cord compression, with 95 % of those dogs having multiple sites of compression. Perhaps this is associated with the role that ligamentum flavum hypertrophy plays in decreasing the diameter of the vertebral canal as well as resulting in compression of the spinal cord. Overall, 64 % of dogs with multiple affected sites had ligamentum flavum hypertrophy. Ligamentum flavum hypertrophy causing spinal cord compression has been previously observed in dogs with DA-CSM [10, 12]. A moderate correlation was observed between the presence of ligamentum flavum hypertrophy and the number of intervertebral disc protrusions, but a reason for why these would occur in only some locations where intervertebral disc degeneration or protrusion is present is unknown. Perhaps this is a reflection of biomechanical changes in a specific site as well as the effect of age and increasing number of degenerated intervertebral discs. The fact that practically half (49 %) of dogs 6 years of age or older had ligamentum flavum hypertrophy does seem to suggest that age may play a role in this finding.

Almost all dogs (98 %) had intervertebral disc degeneration, with a higher prevalence of partial intervertebral disc degeneration. Also, most (80 %) dogs with a single affected site were younger than 6 years of age while the majority (53 %) of dogs with multiple sites affected were 6 years or older. This was not unexpected since most dogs in the study population were middle-aged or older dogs [10, 12, 13]. Statistically, there was a moderate correlation between age and number of protruded discs, as well as degenerated discs.

As for vertebral endplate changes, most dogs had hypointense changes in T1W and T2W images, which is considered indicative of sclerosis, while few dogs had hyperintense changes on T1W and T2W images, suggestive of fatty infiltration [27].

Foraminal stenosis was also prevalent, with some degree of stenosis observed in 81 % of the dogs in this study. A weak correlation was noted between tetraparesis and foraminal stenosis, which may suggest an association between these two, however, there was no correlation between tetraparesis and maximum severity of foraminal stenosis.

The high percentage (89 %) of sites with degenerative changes of the articular process joints was an unexpected finding, since all these dogs had purely disc-associated CSM and no proliferative changes in the articular processes, which are more commonly associated with degenerative changes of the articular processes. A strong correlation was observed between the number of intervertebral discs with total degeneration and number of articular processes with irregular surface and signs of subchondral sclerosis. Interestingly, a previous study observed a weak correlation between intervertebral disc degeneration and degenerative changes of the articular synovial fluid, but not with surface changes; however, that study was done using a comparatively smaller population of 13 Great Danes, where the majority (12/13) had spinal cord compression due to articular process changes. Another difference, likely secondary to breed and type of CSM, was the median age of dogs in that study was 3.9 years [22]. While it is difficult to make a proper comparison between that study and ours, it is interesting to note that both found a correlation between changes in articular process joints and intervertebral disc degeneration. It is unclear how several of these degenerative changes interact and develop, and which would be the primary determining factor. The authors believe that a confluence of anatomical, degenerative, and biomechanical changes are interwoven in the pathogenesis of the investigated changes in DA-CSM.

The main limitation of this study is the lack of an equivalent population of dogs within a similar age group with magnetic resonance imaging of the cervical spine and no signs of CSM for comparison. Other limitations include varying imaging protocols and neurologic examination performed by different neurologists; however, they were all performed by board-certified neurologists at the time of presentation and all images were reviewed by the same person for this study to reduce variations in all subjective observations. A limitation was also the lack of a radiologist in evaluating the images; however, the neurologist in charge of evaluating the MRI images has extensive clinical and research experience with MRI studies in cervical spondylomyelopathy. Also, because it was a retrospective study, extension/flexion MRI studies were not available for all dogs to investigate the dynamic nature of the lesions, and some lesions could be worse than seen on neutral images. Another limitation would be the small number of dog breeds other than Doberman Pinscher, but this most likely represents the distribution of dogs with DA-CSM since the dogs herein presented were selected from a large cohort at the authors’ institution. The aforementioned limitations could be resolved with a prospective study, but it would be difficult to gather a large number of affected and unaffected dogs of all breeds with MRI images for comparison.

## Conclusions

As expected, most dogs with DA-CSM were older Dobermans, though 8 % of dogs were younger dogs (< 3 years). Compressive lesions were typically located in the caudal cervical vertebral region (C6-7, C5-6). Many dogs had concomitant changes such as ligamentum flavum hypertrophy and intervertebral foraminal stenosis. A positive correlation was observed between severity of spinal cord compression and neurologic grade; however, multilevel spinal cord compression was not associated with more severe neurologic signs. Unexpectedly, a very high percentage of dogs with purely DA-CSM had degenerative changes in the articular processes. A possible biomechanical or genetic relationship between degenerative changes in articular processes, ligamentum flavum, and intervertebral discs warrants further investigation.

## Methods

Records were searched from January 2005 to August 2019 for dogs with a confirmed diagnosis of cervical spondylomyelopathy based on clinical and magnetic resonance imaging (MRI) findings in a single academic center. Criteria for being included in the present study were a diagnosis of CSM caused by intervertebral disc protrusion (with or without ligamentum flavum hypertrophy) based on neurologic signs and MRI findings and availability of clinical data and MRI for review. If on initial review the diagnosis of CSM included any other possible intramedullary or extramedullary disease, these cases were immediately excluded. Specific to the diagnosis of CSM, the exclusion criteria were the presence of concomitant spinal cord or nerve root compression caused by osseous proliferation of the articular facets (with or without ligament hypertrophy), or by laminar thickening (with or without ligament hypertrophy). Dogs were also excluded if clinical signs resulted solely from ligamentum flavum hypertrophy or intervertebral foraminal stenosis without concomitant intervertebral disc protrusion.

Data obtained from medical records included breed, age, gender, duration of clinical signs (acute when < 1 week, chronic when ≥ 1 week before diagnosis), presence of ataxia and/or paresis, presence of cervical hyperesthesia, neurologic grade (1 to 5), and presenting complaint. The neurologic grading system was adapted from a previously published system [9]: grade 1, cervical hyperesthesia only; grade 2, mild pelvic limb ataxia or paresis ± thoracic limb involvement; grade 3, moderate pelvic limb ataxia or paresis ± thoracic limb involvement; grade 4, marked pelvic limb ataxia or paresis with thoracic limb involvement; grade 5, tetraparesis with inability to stand or walk without assistance.

Magnetic resonance images were acquired using a high-field, 3.0 Tesla MRI scanner (Achieva 3.0 Tesla, Philips Healthcare) and a surface coil. Dogs were positioned in dorsal recumbency with the cervical region extended in neutral position. Images were acquired using a turbo spin-echo technique. Overall, these were the minimum protocol used: sagittal and transverse T1-weighted and T2-weighted images were obtained. Repetition time (TR) and echo time (TE) were as follows: T1-weighted, TR = 650 ms, TE = 8 ms; T2-weighted, TR = 4000 ms, TE = 120 ms. The number of acquisitions was 2. The flip angle was set at 90°. The field of view was 20 cm with matrix dimensions of 200 × 192 mm. Slice thickness was set at 3 mm with no interslice interval. The entire cervical vertebral region was included (C1-T2) in the sagittal images.

Magnetic resonance images were reviewed by a board-certified neurologist with extensive expertise and experience in evaluating MRI studies of dogs with CSM. The following data was recorded: location of compressive lesions, main compressive lesion (site with the greatest reduction in cross-sectional area and T2-weighted hyperintensity when present), severity of the compression (grade 1 = mild: less than 25 % of the diameter of the spinal cord; grade 2 = moderate: 25–50 %, grade 3 = severe: greater than 50 %), presence of intervertebral disc degeneration, presence and characterization of spinal cord signal changes on T1 and T2-weighted images, presence of ligamentum flavum hypertrophy, intervertebral foraminal stenosis, signs of articular process degenerative changes, presence of spondylosis, and presence of spinal cord atrophy. These changes were classified according to previously published studies [3, 9, 15, 22, 28, 29]. For differentiating intervertebral disc protrusions and extrusions, previously published guidelines were used [30]. Articular process joints were evaluated as a pair per location (C2-3 through C7-T1). The appearance of the intervertebral discs was classified as normal (Pfirrmann grade I), partial degeneration (grade II or III), or total degeneration (grade IV or V) [31]. Changes to the vertebral endplates were also characterized as hyperintense or hypointense in T1- and/or T2-weighted images following previously published criteria [27].

An association was investigated between age, gender, severity of neurologic signs (neurologic grade), duration of clinical signs, history (chronic or acute), main site of spinal cord compression, number of intervertebral discs with signs of degeneration, number of intervertebral disc protrusions, presence of spondylosis, presence of spinal cord signal changes, presence and severity of intervertebral foraminal stenosis, presence of signs of articular process degenerative changes, and presence of ligamentum flavum hypertrophy at the same site of main compression.

Dogs with single vs. multiple sites of spinal cord compression were also compared for: age, gender, severity of neurologic signs (neurologic grade), duration of clinical signs, history (chronic or acute), number of intervertebral discs with signs of degeneration, number of intervertebral disc protrusions, presence of ligamentum flavum hypertrophy, presence of spondylosis, presence of spinal cord signal changes, presence and severity of intervertebral foraminal stenosis, presence of signs of articular process degenerative changes.

For statistical analyses, dogs were also grouped according to neurologic grade as follows: grouped neurologic grade A included dogs graded from 1 to 3 and grouped neurologic grade B included dogs graded 4 and 5. Groups A and B were then compared for the same characteristics as the comparison between non-grouped neurologic grades.

Data were analyzed using SAS statistical software (version 9.4, SAS Institute Inc., Cary, NC). Descriptive statistics were calculated using Proc FREQ, and mean, median and range were reported. Association between listed variables were tested using chi-squared test of independence or Fisher’s exact test as appropriate and Spearman’s rank correlation coefficient (*r*) was used to calculate the relationship between variables. Correlation strength were classified as values < 0.19 considered very weak correlation, 0.2 to 0.39 weak, 0.4 to 0.59 moderate, 0.6 to 0.79 strong and > 0.8 very strong correlation. Positive values indicate that when one variable increases, so does the value of the other variable. The variables of interest were considered significant if *p* < 0.05.

## Data Availability

The datasets used and/or analyzed during the current study are available from the corresponding author on reasonable request.

## Abbreviations

CSM: Cervical spondylomyelopathy
DA-CSM: Disc-associated cervical spondylomyelopathy
MRI: Magnetic resonance imaging
SCSC: Spinal cord signal changes
T1W: T1-weighted
T2W: T2-weighted
